# Real‐World Patterns of Botulinum Toxin Treatment in Hyperkinetic Movement Disorders: A 9‐Year Nationwide Analysis in France

**DOI:** 10.1002/mdc3.70650

**Published:** 2026-05-24

**Authors:** Marion Simonetta‐Moreau, Pierre Karam, Anne Forestier, Bertrand Degos

**Affiliations:** ^1^ Department of Neurosciences University Hospital of Toulouse France, INSERM, UMR1214 Toulouse France, Health Department of Toulouse University Toulouse France; ^2^ PKCS Ecully France; ^3^ Ipsen Paris France; ^4^ Neurology Department, Avicenne Hospital, APHP Hôpitaux Universitaires de Paris‐Seine Saint Denis (HUPSSD), Sorbonne Paris Nord, NS‐PARK/FCRIN Network Bobigny France; ^5^ Center for Interdisciplinary Research in Biology, Collège de France, CNRS UMR7241/INSERM U1050 Université PSL Paris France

**Keywords:** botulinum toxin, dystonia, hyperkinetic movement disorders, myoclonus, tremor

## Abstract

**Background:**

Hyperkinetic movement disorders, including dystonia, tremor, and myoclonus, are disabling conditions often managed with botulinum toxin type A (BoNT‐A). Real‐world evidence on treatment patterns remains limited.

**Objective:**

This nationwide, population‐based study aimed to evaluate trends in BoNT‐A use in France between 2015 and 2023, using the French National Hospital Discharge Database (*Programme de Médicalisation des Systèmes d'Information*, PMSI).

**Methods:**

Patients were classified into four categories (dystonia, tremor, myoclonus, and other abnormal movements), based on International Classification of Diseases, 10th Revision (ICD‐10). Annual changes in the number of BoNT‐A injections and treated patients were analyzed. Treatment adherence and reinjection intervals were also evaluated.

**Results:**

A total of 51,861 patients received BoNT‐A therapy. Dystonia was the predominant indication (44,913; 86.6%). Tremor accounted for 3690 patients (7.1%), other abnormal movements for 2423 (4.7%), and myoclonus for 835 (1.6%). Between 2015 and 2023, BoNT‐A injections increased from 49,829 to 53,828 (+8.0%) and treated patients from 20,023 to 21,489 (+7.3%). While dystonia showed minimal growth in patient numbers (+0.6%), tremor and myoclonus increased substantially (+132.1% and +179.7%, respectively). Repeat BoNT‐A injections were highest in dystonia, with 71.8% of patients receiving ≥3 injections and 57.9% receiving ≥5 injections in total. Median reinjection intervals ranged from 112 days in dystonia to 133 days in myoclonus.

**Conclusions:**

Dystonia remains the leading indication for BoNT‐A in France, with high treatment adherence. The marked growth in tremor and myoclonus underscores the expanding therapeutic role and diversification of BoNT‐A use across hyperkinetic movement disorders.

Hyperkinetic movement disorders encompass a heterogeneous group of neurological conditions characterized by the presence of abnormal involuntary movements, comprising most notably tremor, dystonia, and myoclonus.^1^, ^2^ These disorders, which can be isolated or associated with other neurological or non‐neurological signs, are often disabling and can significantly impair quality of life.^3^ Despite differences in clinical presentation and pathophysiology, overlapping features can complicate their diagnosis and management.^4^

Dystonia is marked by sustained or intermittent muscle contractions causing abnormal, often repetitive movements or postures.^5^ Common focal dystonias include cervical dystonia, facial dystonia (blepharospasm, oromandibular), dystonia of the upper and lower limbs, and laryngeal dystonia.^5^, ^6^, ^7^ Tremor involves rhythmic oscillatory movements produced by alternating muscle contractions.^1^ Myoclonus is a sudden, brief, jerky, shock‐like movement that can affect the extremities, face, or trunk.^8^

Owing to its efficacy and favorable safety profile, botulinum toxin type A (BoNT‐A) has become a cornerstone in the management of hyperkinetic movement disorders, particularly dystonia, tremor, and myoclonus.^6^, ^7^, ^9^, ^10^ Although hyperkinetic movement disorders also include other conditions such as chorea, BoNT‐A is primarily used in clinical practice for the treatment of these disorders. BoNT‐A acts at the cholinergic presynaptic nerve terminal by cleaving the synaptosomal‐associated protein of 25 kDa (SNAP‐25). Ultimately, this inhibits the release of acetylcholine, which in turn prevents muscle contraction and results in local weakness and paralysis.^9^ BoNT‐A is considered the first‐line treatment for focal dystonias, and is increasingly used in other movement disorders to improve function, reduce pain, and enhance quality of life.^6^, ^7^, ^9^, ^10^

There is a generally accepted standard of at least 12 weeks between two BoNT‐A injections.^6^ However, in real‐world practice, intervals may vary depending on the patient's clinical response, tolerability, and logistical factors. To better understand the real‐world use of BoNT‐A outside controlled clinical trials, this nationwide, population‐based study aimed to analyze trends in BoNT‐A therapy in France between 2015 and 2023. Using the French National Hospital Discharge Database (*Programme de Médicalisation des Systèmes d'Information*, PMSI) covering the entire French population, we examined patient characteristics, clinical indications, and injection intervals among individuals with hyperkinetic movement disorders, including dystonia, tremor, and myoclonus, to provide a comprehensive overview of BoNT‐A use in routine neurological care.

## Methods

### Data Source and Study Design

An observational, retrospective study was conducted from January 1, 2015 to December 31, 2023, based on the PMSI database. As previously described, PMSI is a comprehensive claims database that includes standardized discharge summaries for all inpatients and outpatients in public hospitals and inpatients in private hospitals across France.^11^, ^12^, ^13^ Diagnoses are coded according to the 2025 version of the International Classification of Diseases, 10th Revision (ICD‐10), as primary (main reason for hospitalization), related (linked to the primary diagnosis), or significantly associated (comorbidities or complications). Medical procedures are coded using the French Common Classification of Medical Procedures (*Classification Commune des Actes Médicaux*, CCAM).

The study was conducted in accordance with the Declaration of Helsinki and Good Clinical Practice guidelines. Data access was approved by the French Data Protection Agency (*Commission Nationale de l'Informatique et des Libertés*, CNIL). Ethical approval and informed consent were not required, as the study used only anonymized data and did not involve direct patient participation. This study was reported in accordance with the Strengthening the Reporting of Observational Studies in Epidemiology (STROBE) guidelines for observational studies. The completed STROBE checklist is provided in Supplementary Table S1.

### Patient Population

Patients were divided into four groups of hyperkinetic movement disorders, according to ICD‐10 codes and clinical characteristics (Table 1). These groups included: (1) dystonia, encompassing all G24 subcodes; (2) tremor, comprising essential (G25.0), drug‐induced (G25.1), other specified (G25.2), and unspecified tremors (R25.1); (3) myoclonus (G25.3); and (4) other abnormal movements (R25.0 and R25.8). This classification allowed structured analysis of different movement disorder subtypes within the PMSI database. The four groups were not mutually exclusive; hence, a single patient could be assigned to more than one group if coded with multiple conditions.

**TABLE 1:** ICD‐10 codes for hyperkinetic movement disorders

ICD‐10 code	Condition
1. Dystonias	
G24.0	Drug‐induced dystonia
G24.1	Genetic torsion dystonia
G24.2	Idiopathic nonfamilial dystonia
G24.3	Spasmodic torticollis (cervical dystonia)
G24.4	Idiopathic orofacial dystonia (oromandibular dystonia)
G24.5	Blepharospasm
G24.8	Other dystonia
G24.9	Dystonia, unspecified
2. Tremors	
G25.0	Essential tremor
G25.1	Drug‐induced tremor
G25.2	Other specified forms of tremor
R25.1	Tremor, unspecified
3. Myoclonus	
G25.3	Myoclonus
4. Other abnormal movements	
R25.0	Abnormal head movements
R25.8	Other abnormal involuntary movements

### Botulinum Toxin Therapy

Within each study group, we identified patients treated with intramuscular BoNT‐A injections between 2015 and 2023, using the CCAM codes “BALB001,” “PCLB002,” and “PCLB003.” These codes represent a single session of BoNT‐A injections, with BALB001 indicating palpebral injections, PCLB002 indicating intramuscular injections without electromyographic guidance, and PCLB003 indicating intramuscular injections with electromyographic guidance. Patients treated with BoNT‐A were further analyzed according to age, sex, and type of healthcare facility, including regional university hospitals, general or local hospitals, private not‐for‐profit hospitals (*établissements de santé privés d'intérêt collectif*, ESPIC), private hospitals, and other facility types. We assessed the annual progression in both the number of BoNT‐A injections and the number of treated patients across the four groups from 2015 to 2023. Treatment adherence was evaluated by identifying patients who received at least three BoNT‐A injections in total during the study period. Additionally, we analyzed the interval between two injections.

### Statistical Analyses

The four patient groups were analyzed both separately and collectively. Descriptive statistics were used to summarize the data, with categorical variables presented as counts and percentages, and continuous variables as mean ± standard deviation (SD) or median with interquartile range (IQR). No imputation was applied for missing data. All analyses were performed using SQL Server software (version 2022; Microsoft, Redmond, WA, USA).

## Results

### Patient Characteristics

Between 2015 and 2023, a total of 51,861 patients with hyperkinetic movement disorders received at least one BoNT‐A injection in France. The vast majority were treated for dystonia (44,913 patients; 86.6%), with a clear female predominance (64.0% women versus 36.0% men). Tremor was managed with BoNT‐A in 3690 patients (7.1%), again more frequently in women (58.3% versus 41.7%). Other abnormal movements accounted for 2423 patients (4.7%), with women representing 57.5% of cases and men 42.5%. By contrast, myoclonus was treated with BoNT‐A in 835 patients (1.6%), and was the only condition showing a male predominance (56.5% versus 43.5%). The age at the first BoNT‐A injection differed across disorders. Patients with dystonia had a mean ± SD age of 57.4 ± 19.4 years and a median age of 61 years (IQR, 46–71). Those with tremor were older, with a mean ± SD age of 62.2 ± 17.2 years and a median of 66 years (IQR, 53–74). In contrast, patients with other abnormal movements were younger, with a mean ± SD age of 47.1 ± 21.4 years and a median of 50 years (IQR, 32–63). Similarly, myoclonus affected a younger population, with a mean ± SD age of 48.2 ± 22.6 years and a median of 52 years (IQR, 32–66).

### Botulinum Toxin Treatment Trends

The total number of BoNT‐A injections administered for hyperkinetic movement disorders steadily increased by 8.0%, from 49,829 in 2015 to 53,828 in 2023 (Figs. 1A and 2A). There was a modest 2.5% rise for dystonia, from 46,933 to 48,083 injections (Fig. 2B), whereas other disorders showed much stronger growth. Injections for tremor increased from 1392 to 3304 (137.4%), for other abnormal movements from 1235 to 1846 (49.5%), and for myoclonus from 269 to 595 (121.2%) (Fig. 1A). In 2023, most BoNT‐A injections for dystonia were administered in regional university hospitals (52.6%), followed by general or local hospitals (29.9%), private not‐for‐profit hospitals (ESPIC, 12.9%), and private hospitals (4.4%). Similar patterns were seen for other conditions (Fig. 3).

**Figure 1:**
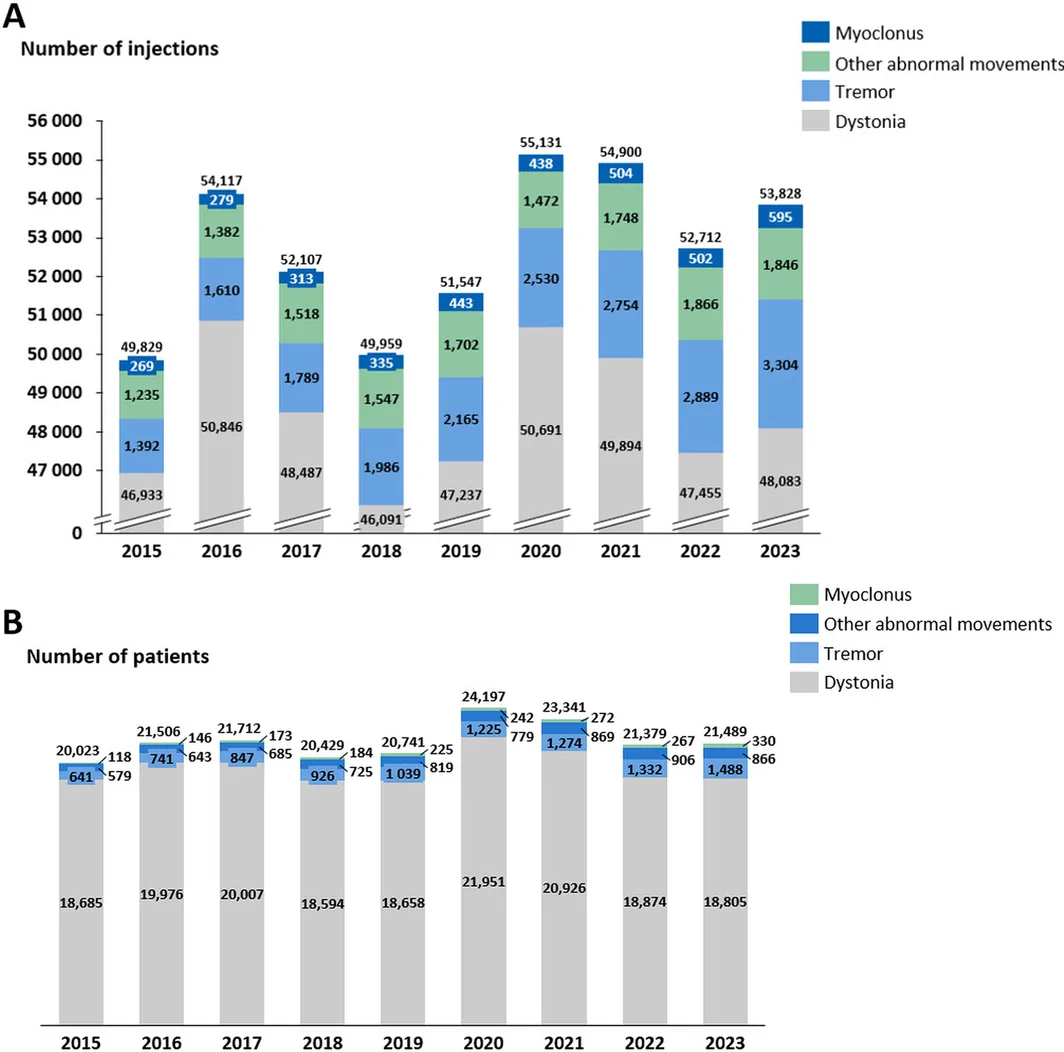
Changes between 2015 and 2023 in the number of botulinum toxin type A (BoNT‐A) injections (A) and in the number of patients with hyperkinetic movement disorders treated with BoNT‐A (B).

**Figure 2:**
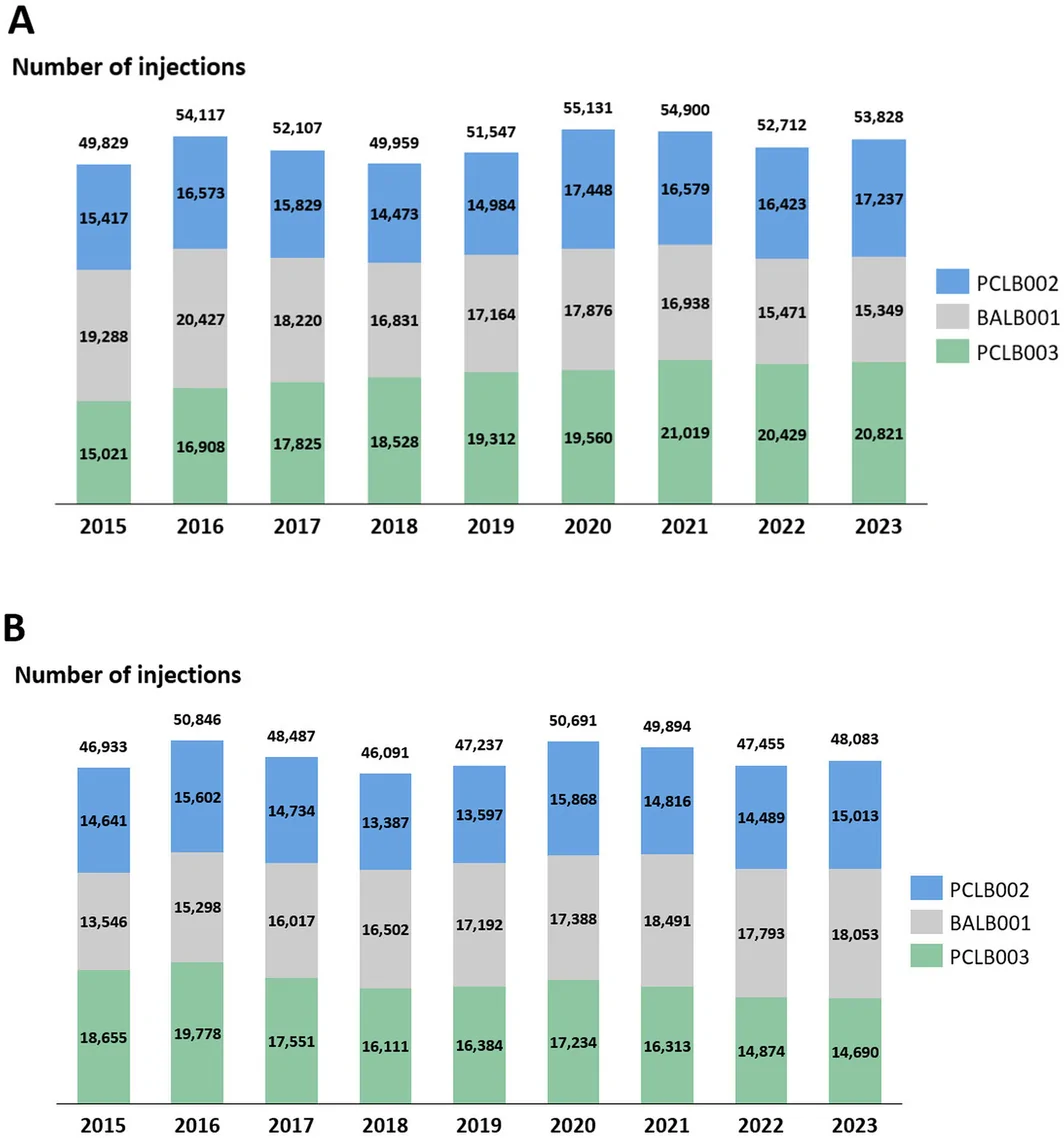
Changes between 2015 and 2023 in the number of botulinum toxin type A (BoNT‐A) injections in the overall study population (A) and in patients with dystonia (B), according to the French Common Classification of Medical Procedures (CCAM) code. BALB001 indicates palpebral BoNT‐A injections, PCLB002 intramuscular injections without electromyographic guidance, and PCLB003 intramuscular injections with electromyographic guidance.

**Figure 3:**
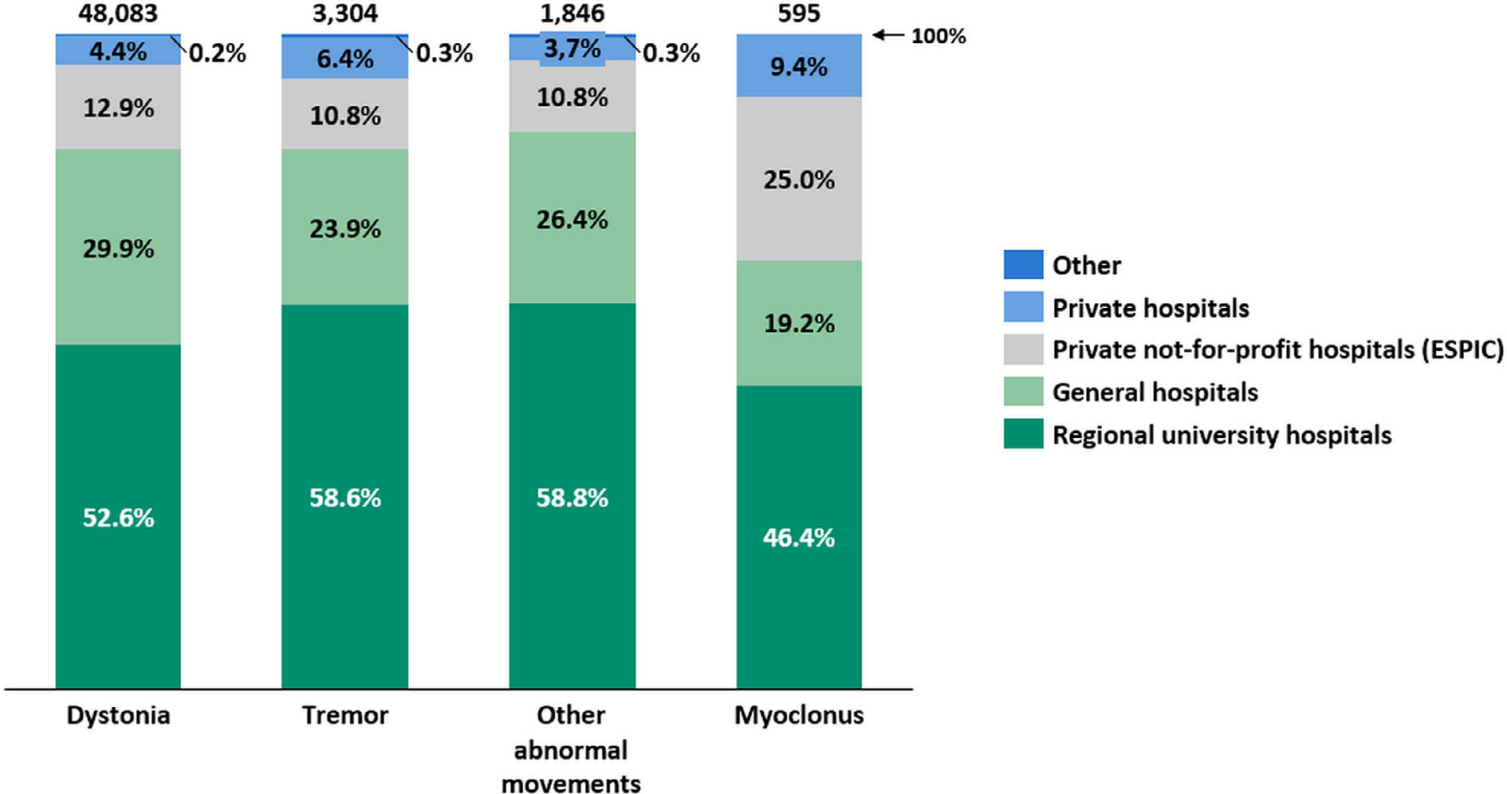
Distribution of BoNT‐A injections by healthcare setting in 2023. ESPIC: Établissements de santé privés d'intérêt collectif.

Similarly, the number of patients receiving BoNT‐A treatment increased from 20,023 in 2015 to 21,489 in 2023, representing a 7.3% overall rise. The increase was small for dystonia, from 18,685 to 18,805 patients (0.6%), whereas tremor showed a strong growth, from 641 to 1488 patients (132.1%) (Fig. 1B). For BoNT‐treated patients with myoclonus, their number increased from 118 in 2015 to 330 in 2023, representing a 179.7% increase (Table 2).

**TABLE 2:** Changes between 2015 and 2023 in the number of patients treated with botulinum toxin type A injections, by International Classification of Diseases, 10th revision (ICD‐10) code

	ICD‐10 code	Condition	2015	2016	2017	2018	2019	2020	2021	2022	2023	Percentage change from 2015 to 2023
Dystonias	G24.0	Drug‐induced dystonia	65	68	77	79	101	89	80	72	99	+52.3%
	G24.1	Genetic torsion dystonia	139	156	170	176	202	229	237	219	228	+64.0%
	G24.2	Idiopathic nonfamilial dystonia	329	333	323	306	302	365	345	340	333	+1.2%
	G24.3	Spasmodic torticollis (cervical dystonia)	4705	4981	4892	4415	4422	5248	4969	4393	4418	−6.1%
	G24.4	Idiopathic orofacial dystonia (oromandibular dystonia)	890	980	1046	932	877	1161	905	784	813	−8.7%
	G24.5	Blepharospasm	7188	7544	7261	6482	6329	7728	6866	5876	5622	−21.8%
	G24.8	Other dystonia	3983	4405	4640	4550	4704	5309	5516	5229	5312	+33.4%
	G24.9	Dystonia, unspecified	1386	1509	1598	1654	1721	1822	2008	1961	1980	+42.9%
Tremors	G25.0	Essential tremor	310	356	434	478	545	684	681	688	782	+152.3%
	G25.1	Drug‐induced tremor	11	11	13	12	15	12	14	18	18	+63.6%
	G25.2	Other specified forms of tremor	157	193	209	237	252	277	302	332	351	+123.6%
	R25.1	Tremor, unspecified	163	181	191	199	227	252	277	294	337	+106.7%
Myoclonus	G25.3	Myoclonus	118	146	173	184	225	242	272	267	330	+179.7%
Other abnormal movements	R25.0	Abnormal head movements	220	252	282	298	352	330	364	388	316	+43.6%
	R25.8	Other abnormal involuntary movements	359	391	403	427	467	449	505	518	550	+53.2%

BoNT‐A treatment adherence was higher among patients with dystonia compared with those treated for other abnormal movements. Overall, 71.8% of patients with dystonia received at least three BoNT‐A injections in total, compared with 49.6% of patients treated for other abnormal movements. When considering a higher threshold of at least five injections in total, 57.9% of patients with dystonia remained on BoNT‐A treatment (Fig. 4). The median interval between two BoNT‐A injections varied by disorder. Patients with dystonia had the shortest median interval of 112 days (IQR, 91–154). Those with other abnormal movements had a median interval of 119 days (IQR, 95–175), while patients with tremor also had a median of 119 days (IQR, 98–168). Patients with myoclonus had the longest median interval of 133 days (IQR, 105–189).

**Figure 4:**
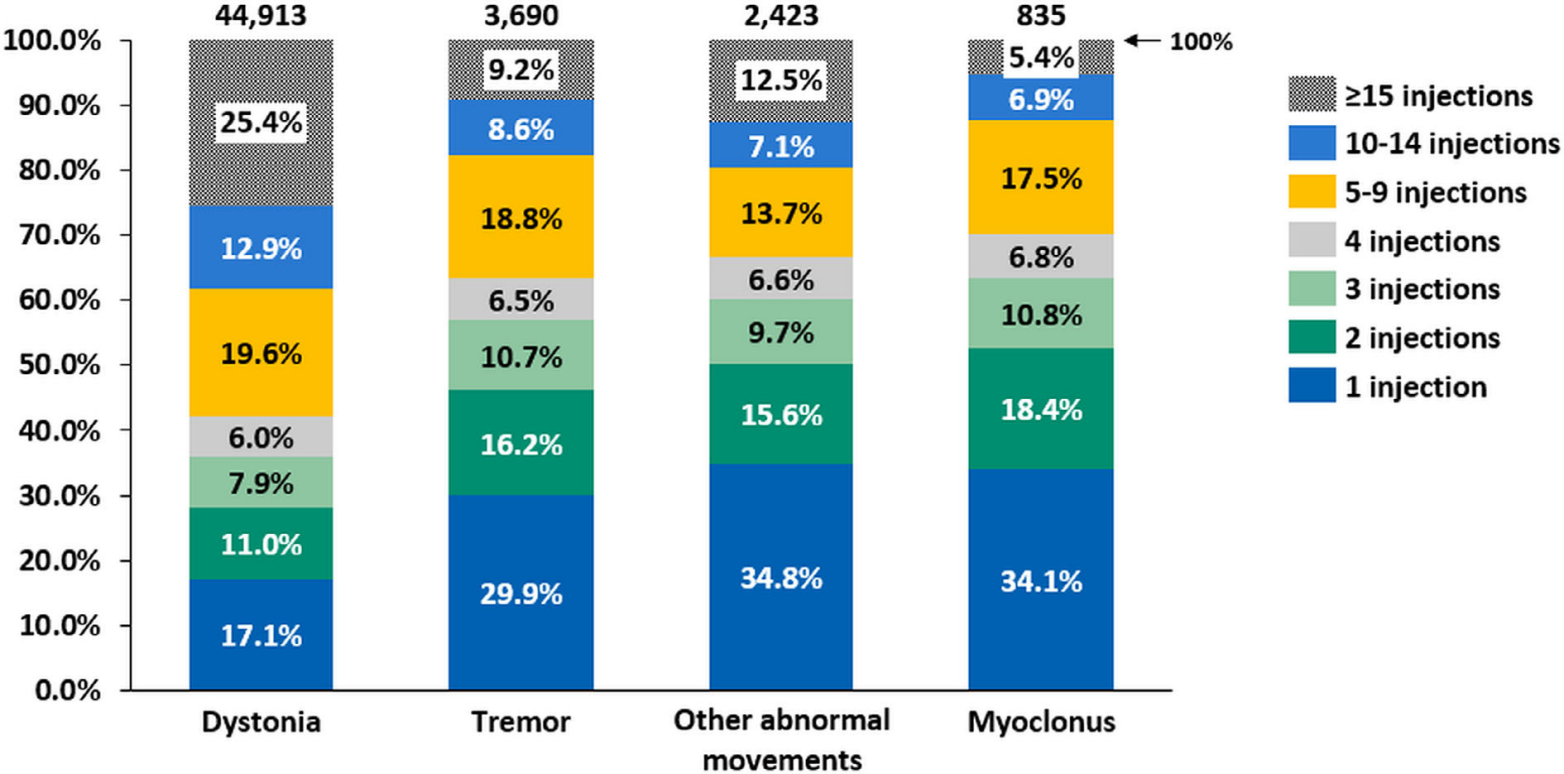
Frequency of botulinum toxin type A injections administered in patients with hyperkinetic movement disorders between 2015 and 2023.

## Discussion

This nationwide, retrospective study provides the most comprehensive real‐world overview to date of BoNT‐A use for hyperkinetic movement disorders in France. Conducted between 2015 and 2023, it confirms that dystonia is the primary indication for BoNT‐A therapy, accounting for 86.6% of all treated patients. The predominance of dystonia (44,913 patients) in our cohort of 51,861 individuals is consistent with the well‐established role of BoNT‐A as the first‐line treatment for focal dystonias, including cervical dystonia, blepharospasm, and oromandibular dystonia.^14^, ^15^, ^16^, ^17^

The efficacy of BoNT‐A in dystonia is supported by a substantial body of evidence. In a Cochrane meta‐analysis of nine randomized, placebo‐controlled trials including 1144 adults with cervical dystonia, a single BoNT‐A treatment session resulted in a clinically relevant reduction of disease‐specific impairment and pain.^15^ Similarly, a meta‐analysis of 26 studies (25 observational and one randomized controlled trial) involving 1103 patients with oromandibular dystonia demonstrated an overall favorable response to BoNT‐A in 96.2% of patients.^17^ For blepharospasm, randomized, placebo‐controlled trials also confirmed that BoNT‐A led to statistically significant and clinically relevant improvements in blepharospasm severity and functional impairment.^16^, ^18^

In our study, patients with dystonia showed high treatment adherence, with 71.8% receiving at least three BoNT‐A injections in total and 57.9% at least five. This pattern of repeated injections reflects the chronic nature of dystonia and indicates that BoNT‐A provides sustained therapeutic benefit in routine clinical practice.^14^ Other studies evaluating BoNT‐A therapy in patients with dystonia also reported an average of 3 to 4 BoNT‐A injections per patient and per year, emphasizing the need for repeated BoNT‐A treatment cycles in this patient population.^17^, ^19^

Patients with dystonia in our study had the shortest median interval between two BoNT‐A injections (112 days or 16 weeks) compared with the other hyperkinetic movement disorders, further suggesting a need for regular retreatment to maintain symptom control.^14^ This 16‐week interval is consistent with previous studies reporting median inter‐injection intervals of 12 to 14 weeks in BoNT‐A‐treated patients with dystonia.^16^, ^19^, ^20^

While the number of BoNT‐A injections and patients treated for dystonia remained stable between 2015 and 2023 (increasing by 2.5% and 0.6%, respectively), reflecting a long‐established treatment paradigm,^14^ we observed a marked rise in the use of BoNT‐A for tremor, myoclonus, and other abnormal movements. The more than doubling in the number of patients treated for tremor (132.1% increase) and myoclonus (179.7% increase) is particularly noteworthy. These observations are consistent with the growing body of evidence from randomized controlled trials supporting the efficacy of BoNT‐A for head and hand tremor, particularly when injections are guided by electromyography.^21^, ^22^ In myoclonus, evidence regarding BoNT‐A remains limited to case reports and small series.^23^, ^24^ Nevertheless, our findings suggest that BoNT‐A is being increasingly recognized as a viable therapeutic option for both tremor and myoclonus, with 53.8% and 47.4% of patients in our study receiving at least three BoNT‐A injections, respectively.

We observed in our study a female predominance in dystonia and tremor, consistent with the known epidemiology of these conditions.^16^, ^17^, ^19^, ^20^, ^22^ By contrast, myoclonus, although representing only a small proportion of BoNT‐A‐treated patients (1.6%), was the only disorder with a male predominance. This observation, while notable, requires further investigation to determine whether it reflects the underlying etiology of myoclonus in the treated population or results from differences in referral patterns and treatment practices. Notably, patients with myoclonus had the longest median interval between two BoNT‐A injections of 133 days or 19 weeks. This longer interval, compared with other assessed hyperkinetic movement disorders, may reflect a more variable disease course or therapeutic response, physician and patient preferences, or the use of BoNT‐A as an adjunctive rather than a primary therapy. Additionally, the underlying phenomenology of myoclonus, brief, jerky contractions involving smaller or less predictable muscle groups, contrasts with the sustained muscle contractions observed in dystonia, which often necessitate more regular BoNT‐A administration for sustained functional benefit.^25^

Our study also revealed consistent patterns in treatment settings, with regional university hospitals accounting for more than half of BoNT‐A injections. This centralization likely reflects the need for specialized expertise in patient selection, injection techniques, and muscle targeting, particularly for complex cases. However, we identified a data gap, with an estimated 5500 BoNT‐A injections missing from private sector records in recent years due to changes in reimbursement schemes in France. This underreporting suggests that the overall use of BoNT‐A, particularly in private practice, is likely higher than captured in our analysis.

Additional methodological limitations should be acknowledged. As an observational and retrospective study, all findings are associative and no causal inferences can be made. Results may also not be generalizable to patient populations outside France. Moreover, reliance on administrative codes carries an inherent risk of misclassification. The accuracy of ICD‐10 codes for identifying neurological conditions in administrative databases has been reported to be suboptimal.^26^ Variability in coding practices, such as the choice of procedure codes (eg, BALB001 for palpebral injections versus PCLB002/003 for intramuscular injections) or differences in guidance techniques (eg, electromyographic versus ultrasound), could introduce additional heterogeneity. In addition, because the four patient groups were not mutually exclusive, individuals with multiple movement disorders may have been counted more than once, although this overlap reflects clinical reality. To address this challenge, we used an algorithm to prioritize, for patients with multiple movement disorders (estimated at 12.6% of patients), the indication associated with the highest number of BoNT‐A injections. Finally, the use of health administrative data precluded access to key clinical details, including injection sites and doses, disease severity, and adverse events.

Despite these limitations, the PMSI database provides a large, representative source of information and contributes valuable evidence to a field where real‐world data remain limited. Taken together, our findings reinforce the central role of BoNT‐A in the management of hyperkinetic movement disorders in France, while also documenting clear trends toward diversification of its indications.

## Conclusions

Conducted between 2015 and 2023, this nationwide, population‐based study of 51,861 patients confirms BoNT‐A as a cornerstone treatment for hyperkinetic movement disorders in France. Dystonia remained the main indication for BoNT‐A treatment, with strong adherence over time. Tremor and myoclonus, though less common, showed the most rapid expansion in use throughout the study period. Reinjection intervals were broadly consistent across indications, typically around 4 months. These real‐world findings underscore BoNT‐A's established role in dystonia and its expanding use in tremor, myoclonus, and other abnormal movements, highlighting its adaptability across diverse movement disorders. The marked growth observed in tremor and myoclonus suggests a gradual broadening of BoNT‐A indications in routine neurological care, which may further shape future treatment strategies for hyperkinetic movement disorders.

## Author Roles

(1) Research Project: A. Conception, B. Organization, C. Execution; (2) Statistical Analysis: A. Design, B. Execution, C. Review and Critique; (3) Manuscript: A. Writing of the First Draft, B. Review and Critique.

M.S.M.: 1A, 1B, 2A, 2C, 3B.

P.K.: 1A, 1B, 1C, 2A, 2B, 2C, 3B.

A.F.: 1A, 1B, 2A, 2C, 3B.

B.D.: 1A, 1B, 2A, 2C, 3B.

## Disclosures

**Ethical Compliance Statement:** Data access was approved by the French Data Protection Agency (*Commission Nationale de l'Informatique et des Libertés*, CNIL). Ethical approval and patient informed consent were not required, as the study used only anonymized data and did not involve direct patient participation. Additionally, we confirm that we have read the Journal's position on the issues involved in ethical publication and affirm that this work is consistent with those guidelines.

**Funding Sources and Conflicts of Interest:** MSM received honoraria from Ipsen for training courses and from AbbVie for lectures. PK is a consultant for Ipsen. AF is an Ipsen employee. BD received honoraria from Ipsen and Merz for presentations and educational events, and from Orion Pharma and Merz for advisory board participation. This study was funded by Ipsen.

**Financial Disclosures for the Previous 12 Months:** None.

## Financial Disclosures and Conflicts of Interest

Author disclosures are available in the Supporting Information.

## Supporting information


**Supplementary Material 1.** Plain language summary.
**Table S1.** STROBE checklist for observational studies.

**Data S1.** Supporting Information.

## Data Availability

Qualified researchers with a valid research question may request anonymized patient‐level data by contacting an Ipsen representative. Further information on Ipsen's Data Sharing Policy is available here: https://www.ipsen.com/science/clinical-trials/clinical-data-transparency/.
