# Supplementary material for: Real‐World Patterns of Botulinum Toxin Treatment in Hyperkinetic Movement Disorders: A 9‐Year Nationwide Analysis in France
Source: Mov Disord Clin Pract. 2026 May 24:10.1002/mdc3.70650. Online ahead of print. doi: 10.1002/mdc3.70650 (PMC13339514; doi:10.1002/mdc3.70650)
Supplement: Supplementary file 1 — Supplementary Material 1. Plain language summary. Table S1. STROBE checklist for observational studies. [file MDC3-9999-0-s001.docx]

**Supplementary Material 1.** Plain Language Summary

Hyperkinetic movement disorders are conditions that cause the body to move too much or in an uncontrolled way. These include dystonia, defined by involuntary muscle contractions; tremor, which is a rhythmic shaking movement; and myoclonus, which is a sudden, brief muscle twitching. These disorders can seriously affect daily life. Botulinum toxin type A (BoNT-A) is an injection treatment that can reduce these abnormal movements. To better understand how BoNT-A is used in the real-world care of hyperkinetic movement disorders, we carried out a nationwide study in France using hospital data from 2015 to 2023. In total, 51,861 patients were treated with BoNT-A. Most had dystonia (44,913; 86.6%), followed by tremor (3,690; 7.1%), other abnormal movements (2,423; 4.7%), and myoclonus (835; 1.6%). Between 2015 and 2023, the number of BoNT-A injections rose from 49,829 to 53,828 (+8.0%) and the number of patients treated with BoNT-A from 20,023 to 21,489 (+7.3%). While patient numbers for dystonia increased only slightly (+0.6%), treatment for tremor (+132.1%) and myoclonus (+179.7%) grew strongly between 2015 and 2023. Patients with dystonia were the most likely to continue treatment, with 71.8% receiving at least 3 injections and 57.9% receiving 5 or more. The average time between two BoNT-A injections ranged from 112 days for dystonia to 133 days for myoclonus. Overall, dystonia remains the main reason for BoNT-A treatment in France, but its use for tremor and myoclonus has grown rapidly. This shows that BoNT-A is becoming an increasingly important and flexible treatment for different movement disorders.

**Supplementary Table S1.** STROBE checklist for observational studies

| **Item No.** | **Recommendation** | **Page No.** | **Relevant text from manuscript** |
| --- | --- | --- | --- |
| **Title and abstract** | | | |
| 1a | Indicate the study’s design with a commonly used term in the title or abstract | Page 1 | Real-world patterns of botulinum toxin treatment in hyperkinetic movement disorders: a 9-year nationwide analysis in France |
| 1b | Provide in the abstract an informative and balanced summary of what was done and what was found | Page 2 | Dystonia remains the leading indication for BoNT-A in France, with high treatment adherence. The marked growth in tremor and myoclonus underscores the expanding therapeutic role and diversification of BoNT-A use across hyperkinetic movement disorders. |
| **Introduction** | | | |
| 2 | Explain the scientific background and rationale for the investigation being reported | Pages 3–4 | Hyperkinetic movement disorders encompass a heterogeneous group of neurological conditions … BoNT-A is considered the first-line treatment for focal dystonias, and is increasingly used in other movement disorders to improve function, reduce pain, and enhance quality of life.^6,7,9,10^ |
| 3 | State specific objectives, including any prespecified hypotheses | Page 4 | To better understand the real-world use of BoNT-A outside controlled clinical trials, this nationwide, population-based study aimed to analyze trends in BoNT-A therapy in France between 2015 and 2023. |
| **Methods** | | | |
| 4 | Present key elements of study design early in the paper | Page 4 | An observational, retrospective study was conducted from 1 January 2015 to 31 December 2023, based on the PMSI database. |
| 5 | Describe the setting, locations, and relevant dates, including periods of recruitment, exposure, follow-up, and data collection | Pages 4–6 | PMSI is a comprehensive claims database that includes standardized discharge summaries for all inpatients and outpatients in public hospitals and inpatients in private hospitals across France.^11-13^ |
| 6a | Give the eligibility criteria, and the sources and methods of selection of participants | Page 5 | Patients were divided into four groups of hyperkinetic movement disorders, according to ICD-10 codes and clinical characteristics (**Table 1**). |
| 6b | Matching criteria (if applicable) | Not applicable | Not applicable |
| 7 | Clearly define all outcomes, exposures, predictors, potential confounders, and effect modifiers. Give diagnostic criteria, if applicable | Pages 5–6 | We assessed the annual progression in both the number of BoNT-A injections and the number of treated patients across the four groups from 2015 to 2023. Treatment adherence was evaluated by identifying patients who received at least three BoNT-A injections in total during the study period. Additionally, we analyzed the interval between two injections. |
| 8 | For each variable of interest, give sources of data and details of methods of assessment (measurement). Describe comparability of assessment methods if there is more than one group | Page 4 | Diagnoses are coded according to the 2025 version of the International Classification of Diseases, 10^th^ Revision (ICD-10), as primary (main reason for hospitalization), related (linked to the primary diagnosis), or significantly associated (comorbidities or complications). Medical procedures are coded using the French Common Classification of Medical Procedures (*Classification Commune des Actes Médicaux*, CCAM). |
| 9 | Describe any efforts to address potential sources of bias | Page 11 | Reliance on administrative codes carries an inherent risk of misclassification. |
| 10 | Explain how the study size was arrived at | Pages 5–7 | Between 2015 and 2023, a total of 51,861 patients with hyperkinetic movement disorders received at least one BoNT-A injection in France. |
| 11 | Explain how quantitative variables were handled in the analyses. If applicable, describe which groupings were chosen and why | Page 6 | Categorical variables presented as counts and percentages, and continuous variables as mean ± standard deviation (SD) or median with interquartile range (IQR). |
| 12a | Describe all statistical methods, including those used to control for confounding | Page 6 | Descriptive statistics were used to summarize the data. |
| 12b | Describe any methods used to examine subgroups and interactions | Page 6 | The four patient groups were analyzed both separately and collectively. |
| 12c | Explain how missing data were addressed | Page 6 | No imputation was applied for missing data. |
| 12d | If applicable, explain how loss to follow-up was addressed | Not applicable | Not applicable |
| 12e | Describe any sensitivity analyses | Not applicable | Not applicable |
| **Results** | | | |
| 13a | Report numbers of individuals at each stage of study—eg numbers potentially eligible, examined for eligibility, confirmed eligible, included in the study, completing follow-up, and analysed | Pages 6–7 | Between 2015 and 2023, a total of 51,861 patients with hyperkinetic movement disorders received at least one BoNT-A injection in France. |
| 13b | Give reasons for non-participation at each stage | Not applicable | Not applicable due to use of administrative database. |
| 13c | Consider use of a flow diagram | Page 20 | Figure 1 |
| 14a | Give characteristics of study participants (eg demographic, clinical, social) and information on exposures and potential confounders | Pages 6–7 | The vast majority were treated for dystonia (44,913 patients; 86.6%) … Similarly, myoclonus affected a younger population, with a mean ± SD age of 48.2 ± 22.6 years and a median of 52 years (IQR, 32–66). |
| 14b | Indicate number of participants with missing data for each variable of interest | Page 6 | No imputation was applied for missing data. |
| 14c | Summarise follow-up time (eg, average and total amount) | Pages 6–8 | The total number of BoNT-A injections administered for hyperkinetic movement disorders steadily increased by 8.0%, from 49,829 in 2015 to 53,828 in 2023 (**Fig. 1A**; **Fig. 2A**). |
| 15 | Report numbers of outcome events or summary measures | Pages 6–8 | There was a modest 2.5% rise for dystonia, from 46,933 to 48,083 injections (**Fig. 2B**), whereas other disorders showed much stronger growth. Injections for tremor increased from 1,392 to 3,304 (137.4%), for other abnormal movements from 1,235 to 1,846 (49.5%), and for myoclonus from 269 to 595 (121.2%) (**Fig. 1A**). |
| 16a | Give unadjusted estimates and, if applicable, confounder-adjusted estimates and their precision (eg, 95% confidence interval). Make clear which confounders were adjusted for and why they were included | Not applicable | Not applicable |
| 16b | Report category boundaries when continuous variables were categorized | Not applicable | Not applicable |
| 16c | If relevant, consider translating estimates of relative risk into absolute risk for a meaningful time period | Not applicable | Not applicable |
| 17 | Report other analyses done—eg analyses of subgroups and interactions, and sensitivity analyses | Pages 6–8 | In 2023, most BoNT-A injections for dystonia were administered in regional university hospitals (52.6%), followed by general or local hospitals (29.9%), private not-for-profit hospitals (ESPIC, 12.9%), and private hospitals (4.4%). Similar patterns were seen for other conditions (**Fig. 3**). |
| **Discussion** | | | |
| 18 | Summarise key results with reference to study objectives | Page 8 | This nationwide, retrospective study provides the most comprehensive real-world overview to date of BoNT-A use for hyperkinetic movement disorders in France. |
| 19 | Discuss limitations of the study, taking into account sources of potential bias or imprecision. Discuss both direction and magnitude of any potential bias | Pages 10–11 | As an observational and retrospective study, all findings are associative and no causal inferences can be made. |
| 20 | Give a cautious overall interpretation of results considering objectives, limitations, multiplicity of analyses, results from similar studies, and other relevant evidence | Pages 11–12 | Taken together, our findings reinforce the central role of BoNT-A in the management of hyperkinetic movement disorders in France, while also documenting clear trends toward diversification of its indications. |
| 21 | Discuss the generalisability (external validity) of the study results | Page 11 | Results may also not be generalizable to patient populations outside France. |
| **Other information** | | | |
| 22 | Give the source of funding and the role of the funders for the present study and, if applicable, for the original study on which the present article is based | Page 13 | This study was funded by Ipsen. |
